# Innovations in multidisciplinary education in healthcare and technology

**DOI:** 10.1007/s40037-015-0186-8

**Published:** 2015-05-29

**Authors:** Joost van Hoof, Elisabeth L.M. Zwerts-Verhelst, Marianne E. Nieboer, Eveline J.M. Wouters

**Affiliations:** 1 Fontys University of Applied Sciences, Fontys EGT–Centre for Healthcare and Technology, Dominee Theodor Fliednerstraat 2, 5631 BN Eindhoven, The Netherlands; 2 Fontys University of Applied Sciences, Institute of Allied Health Professions, Dominee Theodor Fliednerstraat 2, 5631 BN Eindhoven, The Netherlands; 3 Fontys University of Applied Sciences, Institute of Human Resource Management and Psychology, Emmasingel 28, 5611 AZ Eindhoven, The Netherlands

**Keywords:** Health care, Technology, Education, Prototyping, Multidisciplinary

## Abstract

The growing importance of technology in health care calls for interdisciplinary study programmes in which students with various backgrounds work together in exploring and designing new solutions for real-life problems. The Centre of Healthcare and Technology of Fontys University of Applied Sciences (Fontys EGT), the Netherlands, is presented as an example of how new initiatives in the field of education at the crossroads of health care and technology can be shaped and implemented in practice. A case study illustrating one of the student projects is provided as an example of the approach to educational innovation and interdisciplinary collaboration.

## Introduction

The increasing pace at which technological innovations emerge and are piloted and adopted in health care requires a different approach in education at the crossroads of health care and technology. These innovations have a far-reaching impact on the way health care professionals carry out their daily tasks and the way health care organizations support planning of these daily routines. Embracing technology in daily care has also led to a change in the technological sector. This sector develops and designs new technologies and adapts existing technologies for applications in the domain of health care. Successful development and implementation requires not only an interdisciplinary approach to these processes, but also an intensive cooperation with the end-users of such technologies: various types of health care professionals, patients and family carers. Since 2011, Fontys University of Applied Sciences (UAS) has provided education in this field through its Centre for Healthcare and Technology (Fontys EGT) [1]. Fontys EGT and a project example are presented to show how new initiatives in the field of education and research in the domain of care and technology can be shaped in practice.

## Structure and functions

Fontys EGT is an interdisciplinary centre which combines knowledge and expertise of eight institutes within Fontys UAS, namely the Institutes of Allied Health Professions, Nursing, Information and Communication Technology, Engineering, Technology & Logistics, Human Resource Management & Psychology, Sports, and Natural Sciences. The centre is dedicated to educating Bachelor students, mainly through offering a minor (half-year programme) on health care and technology, as well as internships, graduation projects, and so on. The minor programme consists of coursework taught by lecturers from the various participating institutes, excursions and visits to fairs and symposia, and desk research assignments (15 European credits). These provide students with a broad foundation of knowledge, and help students to speak each other’s professional language and bridge cultural gaps. For instance, all students practice drawing blood on artificial arms, perform ultrasounds on each other, and produce electrical circuits together. Students within the interdisciplinary group alternate as experts in different domains. The particular character of the participating institutes is reflected in the overall programme. The amount of courses decreases over time, and the emphasis moves to a group project, in which students work together on making a prototype of a technological solution for a specific health care-related challenge. This solution is designed and tested with end-users (user-centred design). The interaction between students with various backgrounds contributes to the quality of the end-work. Each project is supervised by two lecturers, one with a background in technology, and the other with a background in health care. This allows students to learn from each other’s domain and look at the problem from the two main perspectives. The need for developing mutual communication skills is essential and becomes paramount during the design phase. Testing takes place on both the individual and group level through presentations, products and reports. Testing protocols and documents are also based on a multidisciplinary approach, and are included in the exam protocols of each participating institute. Again, these institutes recognize some of their characteristics in the testing protocols, which are sometimes focused on either skills or knowledge.

## Skills labs

**Fig. 1:**
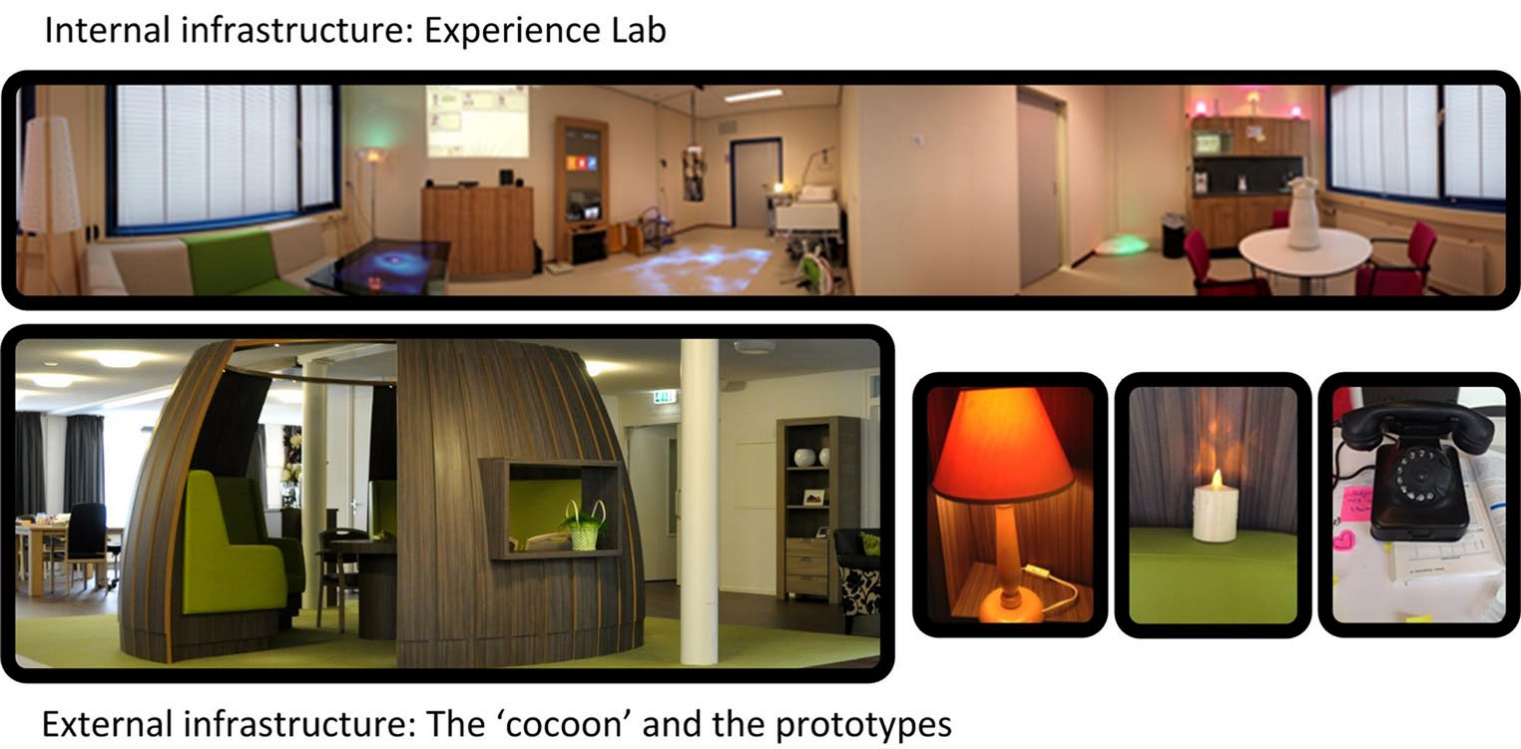
Fontys EGT used both internal infrastructure such as the Experience Lab, as well as infrastructure in the field. One of the partner care organizations hosted the Retrophone project, which encompassed making alterations to the lighting and introducing a reminiscence telephone

The institutes work together on the creation of common skills labs, which try to offer both students and lecturers/researchers tools to learn and educate about health care technologies in an enriched environment. Within Fontys UAS, there are multiple laboratory spaces that are visited as part of the curriculum of Fontys EGT. The Fontys EGT experience lab is an exclusive meeting space for students, in which a number of technologies can be explored and used for home care purposes, including robotics and home automation systems (Fig. 1, top). There is a call centre which can be used for practising e-health. The best prototypes made by students are integrated into the laboratory, and can be developed further. The laboratory, therefore, also serves as a showroom for excellent students, and provides inspiration for new cohorts. The laboratory is run by interns, who coordinate the space and use it as their basis for activities. This physical location was built with the intention to be a challenging, innovative, and inspiring environment where students meet and work together: an incubator for new insights and ideas.

## Project example: Retrophone

The Retrophone project (Fig. 1, bottom) is shown as an example of the multidisciplinary design education of Fontys EGT. As far as possible, projects are connected to on-going research that is being conducted at Fontys UAS, such as the Nursing Home of the Future programme [2, 3]. The Retrophone project encompassed making a product for nursing home residents with dementia that could be used for leisure and as part of a multisensory stimulation programme, building on previous work in this field [4, 5].

In general, there are numerous technological products to stimulate social contacts and activities, but the actual use of these products is often poor, given the complexity or the lack of time and assistance. One example is what we call a ‘cocoon’, a sheltered place in which six people with dementia can sit down and withdraw, which was constructed by a partner nursing home. Although the cocoon seemed promising, it could not be used for practical purposes as the space was uninviting and the technologies too complicated. Therefore, students were asked to develop improvements.

The methodology used was as follows. First, interviews were held with the team leader, the manager, the activity facilitator and a family carer, as well as ten residents with dementia. These interviews yielded information on the use and non-use of the existing infrastructure. Based on the interviews, a taxonomy of design features was made from which personas were developed. Personas are fictional characters used in design processes. Subsequently, a mind mapping session was conducted to come up with design concepts, which were discussed with experts. Two concepts were chosen for further prototyping. These prototypes were tested based on a test plan, encompassing actual user tests and interviews.

The second phase consisted of observations with three residents with dementia. The prototypes concerned making improvements to the existing lighting scheme inside the cocoon in order to increase the homeliness and the sense of security (LED candles were used), as well as the introduction of the Retrophone, an old-fashioned telephone which could be used to ring people and listen to a song (reminiscence). It was important that the participants recognized the telephone and its ring tone, that there was a degree of desired behaviour (i.e., answering the call). After hearing two introduction sentences, participants heard a song, and after the song was finished, there was a closing sentence. Relatives and carers could also use the Retrophone for making actual phone calls. Observations were made of the actual use. Residents valued the homeliness of the lighting intervention, in particular, the LED candles. When testing the Retrophone, the results were favourable. Some of the residents needed some encouragement to answer the phone, after which they engaged in a conversation. Another resident needed some assistance to pick up the horn, and then swayed to the music as a sign of enjoyment. Thereafter, the resident used the Retrophone independently and joined in with the conversation smilingly. In conclusion, the ringtone was recognized by all three users and the level of assistance varied depending on the stage of dementia.

## Do’s for other universities

- Involve lecturers from various faculties both in the development of the curriculum, as well as in supervision of students and lecturing, and develop a shared responsibility for the programme.
- Work on development of a shared vocabulary and set of values: let students experience what it is to be a student of another faculty by exposing them to mutial practicals.
- Let students have contact with target groups for verification of their product. (user-centred design).
- Treat students as experts of their own domain during lectures and design assignments.
- Stimulate the interaction between students and have them work together in a physical space.
